# A Real Valued Neural Network Based Autoregressive Energy Detector for Cognitive Radio Application

**DOI:** 10.1155/2014/579125

**Published:** 2014-10-29

**Authors:** A. J. Onumanyi, E. N. Onwuka, A. M. Aibinu, O. C. Ugweje, M. J. E. Salami

**Affiliations:** ^1^Department of Telecommunication, Federal University of Technology, Minna, Niger State, Nigeria; ^2^Digital Bridge Institute, Abuja, Nigeria; ^3^Department of Mechatronic Engineering, International Islamic University Malaysia, Kuala Lumpur, Malaysia

## Abstract

A real valued neural network (RVNN) based energy detector (ED) is proposed and analyzed for cognitive radio (CR) application. This was developed using a known two-layered RVNN model to estimate the model coefficients of an autoregressive (AR) system. By using appropriate modules and a well-designed detector, the power spectral density (PSD) of the AR system transfer function was estimated and subsequent receiver operating characteristic (ROC) curves of the detector generated and analyzed. A high detection performance with low false alarm rate was observed for varying signal to noise ratio (SNR), sample number, and model order conditions. The proposed RVNN based ED was then compared to the simple periodogram (SP), Welch periodogram (WP), multitaper (MT), Yule-Walker (YW), Burg (BG), and covariance (CV) based ED techniques. The proposed detector showed better performance than the SP, WP, and MT while providing better false alarm performance than the YW, BG, and CV. Data provided here support the effectiveness of the proposed RVNN based ED for CR application.

## 1. Introduction

The energy detector (ED) has been widely proposed for spectrum sensing (SS) in cognitive radio (CR) owing to its design simplicity, fast sensing periodicity, and ability to detect primary user (PU) signal without* a priori* knowledge of its waveform structure (except for the knowledge of noise statistics) [1–11]. However, the demand for fast sensing and high detection accuracy in CR has revealed particular limitations with the ED. In fast sensing, the ED tends to obtain fewer samples for spectral estimation which leads to reduced resolution and invariably poor detection performance [12–15]. Also, it is known that the ED performs poorly in low signal to noise ratio (SNR) conditions and fluctuating noise environments [12–14] which results in increased false alarm rate. These challenges have compelled the need for new improved ED techniques capable of providing better local sensing results at acceptable detection and false alarm rates in both low and high SNR conditions. This serves as the motivation for our research.

Towards developing an ED, several usable spectrum estimation techniques are well-known and these can be broadly divided into parametric and nonparametric techniques. Some examples of these nonparametric techniques include the simple periodogram (SP), Welch periodogram (WP), and the multitaper (MT), while parametric techniques include the Yule-Walker (YW), Burg (BG), and covariance (CV) techniques. Generally, the ED has been widely assumed to be based on the periodogram approach (developed either in time or in frequency domain) and this could be presumably responsible for its poor performance owing to known limitations of the periodogram, for example, large noise variance [13, 14]. On the other hand, few works have been identified on the use of parametric techniques, particularly the autoregressive (AR) technique, in CR [16–22]. Also, these few works have focused more on its use in spectrum hole prediction rather than as an ED for CR application.

In this work, the focus is not on developing a new spectrum estimation technique but rather on proposing the use of a real valued neural network (RVNN) based autoregressive (AR) spectrum estimation technique for ED application in CR. The choice of the RVNN based AR technique was based on its advantage over the popular BG technique in solving the challenge of spectral line splitting at high SNR and large model order conditions [23]. Consequently, it was observed here that such an advantage would lead to an improved ED with less probability of false alarms for CR application. Results of this contribution are presented in Section 5. Also, while authors in [23] developed the RVNN technique and applied it in voice activity detection, in our own work, we propose necessary additions to standardize the approach as a fully functional ED using an empirical threshold estimation technique for CR application. The RVNN based AR model coefficient estimator developed in [23] has never been applied as an ED for CR application; hence, its application was considered here to exploit its inherent advantage. Therefore, once developed as a functional ED, it was tested using simulated data particularly in low and high SNR conditions and varying model order values as described in Section 4 and results obtained with corresponding implications are presented in Section 5. Appropriate conclusions are drawn in Section 6.

## 2. Brief Review of Energy Detector Techniques

**Figure 1:**
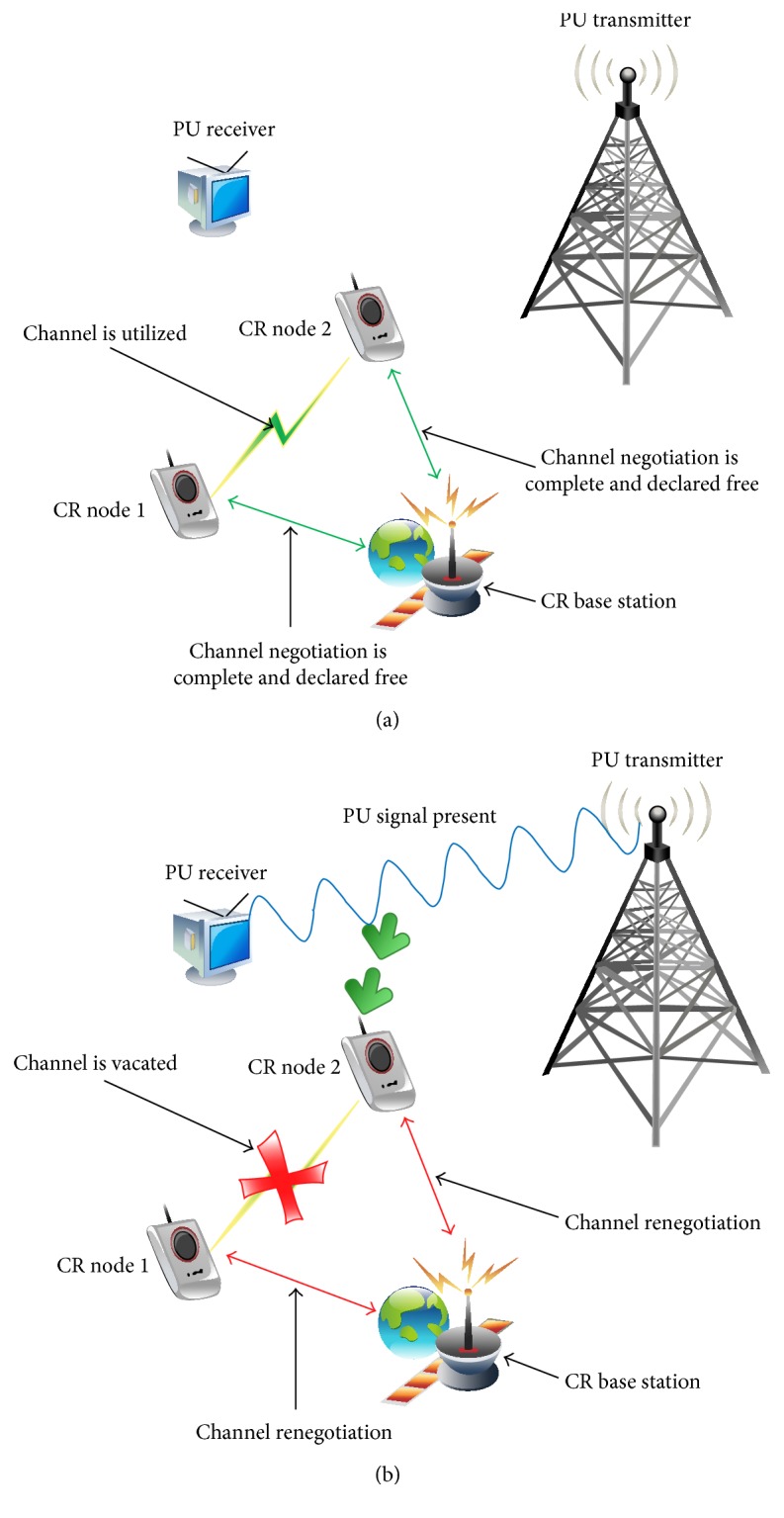
(a) Absence of PU signal permitting CR nodes to negotiate and utilize free band. (b) Presence of PU signal triggers CR nodes to renegotiate and vacate band.

Energy detectors (ED) sense the presence or absence of PU signals by estimating the energy content of a specified band using the power spectral density (PSD) measurements and comparing with predetermined thresholds [12, 14]. This aids the CR device in vacating PU occupied bands to avoid interference and use vacant bands to improve spectrum utilization. Figures 1(a) and 1(b) provide a representation of this process. Towards this goal, signals above the threshold typically signify PU presence, *H*_1_, and below threshold as noise, *H*_0_. These hypotheses *H*_0_ and *H*_1_ are typically represented as

(1) $$\begin{array}{c} H_{0}:x\left(n\right)=w\left(n\right) \end{array}$$

(2) $$\begin{array}{c} H_{1}:x\left(n\right)=s\left(n\right)+w\left(n\right), \end{array}$$

where *w*(*n*) represents samples of additive white Gaussian noise (AWGN), *s*(*n*) denotes samples of PU signal, and *x*(*n*) denotes samples of received signal at the CR input considering a perfect channel.

**Figure 2:**
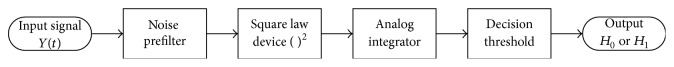
Energy detection in time domain.

**Figure 3:**
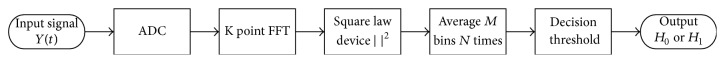
Energy detection using the simple periodogram (frequency domain approach).

EDs are typically developed to decide on these hypotheses either in time or in frequency domain. In time domain, it consists of an input noise prefilter, a magnitude square law device, an integrator, and a detector as shown in Figure 2. This approach has been used in several works [12–15] and well-known for its simplicity and low design and development cost. However, it is based on the super heterodyne technique which is limited by its slow voltage ramping response time [6]. Such a delay might be intolerable for CR operation. Another approach is to realize the ED in the frequency domain using the well-known periodogram as shown in Figure 3. This provides an opportunity to achieve full digitization of the detection process; however, this approach constrains the sensing bandwidth owing to known limitations of typical fast Fourier transform (FFT) realizations [8].

Another method identified for the ED in CR is the Welch periodogram (WP) approach [24]. Particularly, in [25], performance analysis was carried out on the use of WP in Rayleigh fading channels while [26, 27] used WP for OFDM systems in CR. These authors observed that WP operates well for narrowband sensing; however, they conclude that the approach is limited by the excessive variance around the true mean PSD value. This increases the probability of false alarm of the system. Another approach using the Multitaper (MT) technique was proposed in [28] with basic descriptions provided from filter-bank theory point of view. This technique was extended in [29] for signal detection in CR and results were compared with the time domain based ED. It was observed that MT performed better in low SNR conditions under Raleigh fading channels. This was based on the technique's dependence on both magnitude and phase of received samples. Also, [30] provided an improved MT method for CR application in which a recursive method to improve the accuracy of estimates while keeping real-time properties uncompromised was proposed. Authors compared their technique with the SP and ordinary MT and observed better performance for their improved MT.

On the other hand, the use of autoregressive (AR) methods has been sparsely pursued, especially for energy detection in CR. The AR methods present a class of techniques popularly classified as parametric techniques. Though not widely used in CR, we observed that [19–21] proposed AR for spectrum hole detection in CR using time series forecasting. Authors reported on the performance of the proposed approach; however, it remains unclear how well such an approach would perform in typical deployment conditions. Also, authors in [21, 22] used particle and Kalman filtering for spectrum availability forecasting. In [17], authors presented a comparison of SP and Yule-Walker (YW) AR techniques and argued that better performance could be achieved by the YW if large model orders are considered. However, most of these applications have not considered AR application for ED in CR, and hence, it remains unknown how well such techniques would perform if employed. Consequently, we considered the use of a real valued neural network (RVNN) AR model estimator [23] to develop a detector for CR application. This approach provides data reported in [23] which was leveraged upon here to develop an improved ED for CR. Consequently, it is the focus here to develop, evaluate, and analyze its performance for CR application.

## 3. Development of the Real Valued Neural Network Based Energy Detector

### 3.1. An Overview of the RVNN Based Autoregressive Model Estimation Technique

The working principles of the real valued neural network (RVVN) autoregressive (AR) coefficient estimation technique were based on [23]. Thus, we present here only specific details needed to develop the ED for CR application; however, for further details, we refer to [23]. It is known that an AR system driven by white noise *x*(*n*) produces an output sequence *y*(*n*) given as

(3) $$\begin{array}{c} y\left(n\right)=-\overset{P}{\underset{k=1}{\sum }}a_{k}y\left(n-k\right)+b_{0}x\left(n\right), \end{array}$$

where *a*_*k*_, 1 ≤ *k* ≤ *P* and *b*_0_ denotes the model coefficients and *P* is the model order. By taking the *z*-*transform* of (3) and rearranging the terms therein, the transfer function *H*(*z*) of the AR-system can be obtained as

(4) $$\begin{array}{c} H\left(z\right)=\frac{Y\left(z\right)}{X\left(z\right)}=\frac{b_{0}}{1+\sum_{k=1}^{P}a_{k}z^{-k}}. \end{array}$$

By assigning *b*_0_ = 1, *z* = *e* ^−*jw*^ and taking the square of (4), we obtain the SNR as

(5) $$\begin{array}{c} \text{SNR}={\left|\frac{Y\left(jw\right)}{X\left(jw\right)}\right|}^{2}=\frac{1}{{\left|1+\sum_{k=1}^{P}a_{k}e^{-jwk}\right|}^{2}}. \end{array}$$

**Figure 4:**
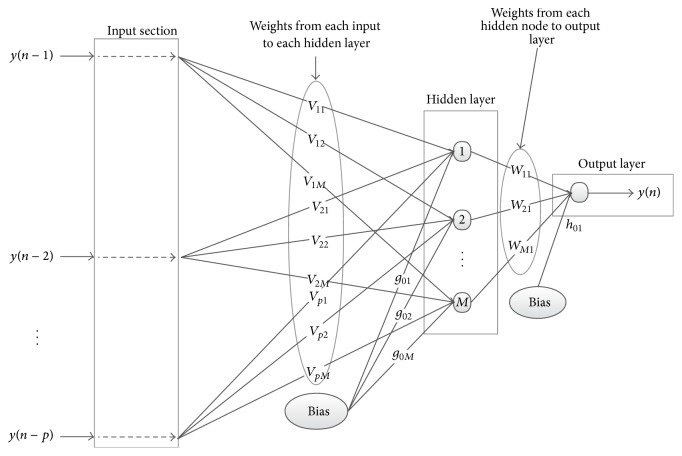
A two-layered RVNN network system.

To estimate the model coefficient *a*_*k*_, we used a two-layered RVNN as shown in Figure 4.

By observing Figure 4, the output sequence of the RVNN system can be obtained as

(6) $$\begin{array}{c} y\left(n\right)=\alpha F\left(\overset{M}{\underset{l=1}{\sum }}w_{l1}\theta_{l}+h_{01}\right), \end{array}$$

where **F** denotes the representation of a linear transfer function, *M* is the number of neurons in the hidden layer, *w*_*l*1_ represents the weight connecting node *l* in the hidden to output layer, *h*_01_ is the bias term of the output neuron, *θ*_*l*_ denotes the output of the hidden node *l*, and *α* is the adaptive coefficient of the linear output activation function. The hidden node output *θ*_*l*_ is obtained as

(7) $$\begin{array}{c} \theta_{l}=\beta_{l}F\left(\overset{P}{\underset{k=1}{\sum }}v_{kl}y\left(n-k\right)+g_{0l}\right), \end{array}$$

where *v*_*kl*_ is the weight connecting input nodes *k* to hidden node *l*, *g*_0*l*_ is the bias of the hidden node, and *β*_*l*_ is the adaptive coefficient of the hidden node linear transfer function. By using linear transfer activation functions in both hidden and output layer, that is, **F**(·) is linear, and substituting (7) into (4), the output sequence is obtained as

(8) y(n)  =αF(∑l=1Mwl1(βlF(∑k=1Pvkly(n−k)+g0l))+h01).

By rearranging terms in (8) and comparing with (4), the model coefficients *a*_*k*_ can be expressed as

(9) $$\begin{array}{c} a_{k}=\alpha \overset{M}{\underset{l=1}{\sum }}w_{l1}\beta_{l}v_{kl}. \end{array}$$

Thus, the AR model coefficients can be estimated from the synaptic weights and coefficients of the adaptive activation function of a properly trained two-layered RVNN. Furthermore, by assuming that the model order *P* is known* a priori*, then the number of neurons in the hidden layer can be determined by using the* priori* data length information at the input. To formulate the necessary constraints for proper evaluation of the system, the solution to a set of linear equations that gives the best performance over the* a priori* fixed model order was obtained. We obtained this by considering a data set of length *N*, for which the required number of training data set *L* was obtained as

(10) $$\begin{array}{c} L=N-P+1. \end{array}$$

The required number of training equations *N*_eq_ is expressed as

(11) $$\begin{array}{c} N_{\text{eq}}=P\times L \end{array}$$

and for *M* hidden nodes, the number of unknowns for the network was obtained as

(12) $$\begin{array}{c} N_{\text{un}}=\left(P+1\right)M+M+1. \end{array}$$

It is known from [23] that the case of *N*_eq_ ≫ *N*_un_ produces better results with respect to the unseen data. Therefore, by putting (11) and (12) into the inequality (*N*_eq_ ≫ *N*_un_), we obtained the optimum number of neurons *M* to satisfy

(13) $$\begin{array}{c} M\ll \frac{\left(P\times L\right)-1}{P+2}. \end{array}$$

With these necessary parameters well established, the algorithm used for the RVNN system is as follows.

(1) We normalized the input data sequence to band-limit the acquired signal using the *Z*-score normalization technique. Other techniques that could be used are the Min–Max, sigmoidal, or unitary data normalization techniques.
(2) The data were formatted using frame blocking and appropriate windowing of data sequencing.
(3) The optimum number of neurons was determined using (13).
(4) The model coefficient was estimated using (9).
(5) Finally, training of the RVNN system was done using the back propagation (BP) supervised learning algorithm.

### 3.2. The RVNN Based Energy Detector

**Figure 5:**
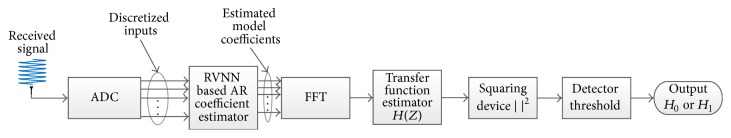
The newly proposed RVNN based ED.

With the RVNN model estimator in place, we developed the RVNN based ED using the proposed schematic of Figure 5. This was achieved by generating the power spectral density (PSD) of the system using (5) and introducing a well-designed detector based on an empirical threshold estimation technique (details of method in Section 4.2). An overview of the design process is as follows.

(1) A simple analogue-digital-converter (ADC) was used for digitization. Here the number of samples was determined using the known Nyquist rate of 8 KHz for a maximum bandspan of 4 KHz for wideband simulation and 100 Hz for narrowband simulation.
(2) The fast Fourier transform (FFT) was used for the frequency response estimation. Appropriate zero padding was employed for cases of fewer samples for FFT operation.
(3) The transfer function estimator was realized using the frequency response vector obtained from appropriate adaptive filtering of the FFT samples.
(4) A simple square law device was introduced for performing the squaring operation.
(5) The detector analyzer was realized using empirically deduced thresholds (details in Section 4.2).
(6) Our proposed detector of Figure 5 can be used for CR application and details of methods used for its analysis are presented in the next section.

## 4. Method of Modelling and Simulation

In this section, we provide details of simulation parameters and analytical models used for evaluating the performance of our proposed detector and other methods for comparison.

### 4.1. Hypothesis Testing

Additive white Gaussian noise (AWGN) with zero mean and unit variance was used and the probability of false alarm *P*_FA_ conditioned for the null hypothesis (1) was obtained using the expression for large time-bandwidth product given in [31]. The *P*_FA_ for noise samples distributed according to a Gaussian variate *N*(2*TW*, 4*TW*) is given as [31]

(14) PFA=18πTW∫VT∞exp⁡[−(y−2TW)28TW]dy

(15) =12erf⁡c[VT−2TW22TW],

where *T* denotes the observation interval, *W* the bandwidth being sensed, *V*_*T*_ the threshold, and *TW* the time-bandwidth product. These were translated appropriately as follows: *TW* is known as the total number of samples *N* (at Nyquist rate), 2*TW* is the sample mean $\hat{\mu }$ of the noise PSD, and 4*TW* is the sample variance ${\hat{\sigma }}^{2}$ of the noise PSD (the noise power). Hence, we rewrite (15) as

(16) $$\begin{array}{c} P_{\text{FA}}=\frac{1}{2}\mathrm{erf}c\left[\frac{V_{T}-\hat{\mu }}{\sqrt{2{\hat{\sigma }}^{2}}}\right]. \end{array}$$

We note that the particular use of (15) according to [31] was based on the condition of a large time-bandwidth product, meaning large sample number. Next, to estimate the probability of detection *P*_*D*_, we considered a modulated discrete signal *S*(*nT*) given as

(17) $$\begin{array}{c} S\left(nT\right)=A\left(t\right)\cos\left(2\pi f_{c}nT\right)\;\forall n=0,1,\ldots ,N-1, \end{array}$$

where *A*(*t*) represents the expression for the modulating signal, *f*_*c*_ the carrier frequency, *N* the total number of samples, and *T* the sampling time.

**Figure 6:**
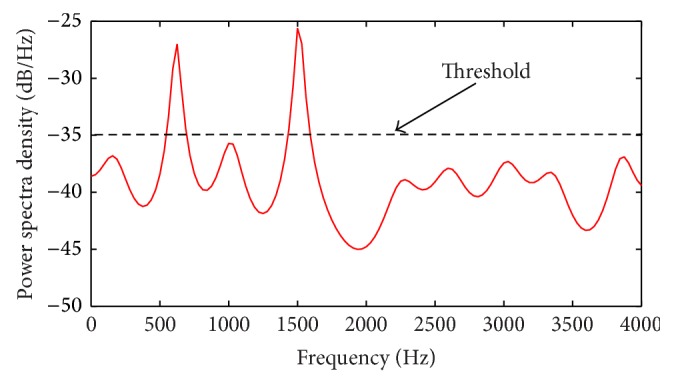
Wideband spectrum sensing of 4 KHz showing two PU signals at 600 Hz and 1.5 KHz in AWGN (occupancy = 10%).

For examination of the effect of wideband sensing, we considered two PU signals with equal amplitudes *A*_1_ = *A*_2_ = 10 dB, and frequencies *f*_*c*1_ = 600 Hz, *f*_*c*2_ = 1500 Hz estimated over a relatively wide sensing span of 4 Hz using a wideband occupancy of 10% as shown in Figure 6. We note that though low frequencies were used, the setup could easily apply to high frequency bands with the same concept being applicable.

**Figure 7:**
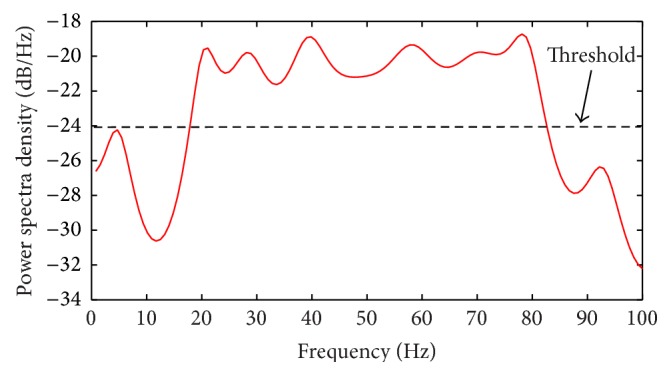
Narrowband spectrum sensing of 100 Hz showing PU bandwidth of 60 Hz and guardbands of 20 Hz on both sides.

For narrowband sensing, a typical PU signal of bandwidth *W* = 60 Hz was simulated for a narrowband sweep of 100 Hz as shown in Figure 7 using the RVNN based ED. These input data were kept constant for all simulation conditions and other EDs compared herein. Then the *P*_*D*_ was estimated using [31]

(18) $$\begin{array}{c} P_{D}=\frac{1}{2}\mathrm{erf}c\left[\frac{V_{T}-2TW-\lambda }{2\sqrt{2}\sqrt{TW+\lambda }}\right], \end{array}$$

where *λ* denotes the SNR for the estimated spectrum.

### 4.2. Threshold Estimation

**Figure 8:**
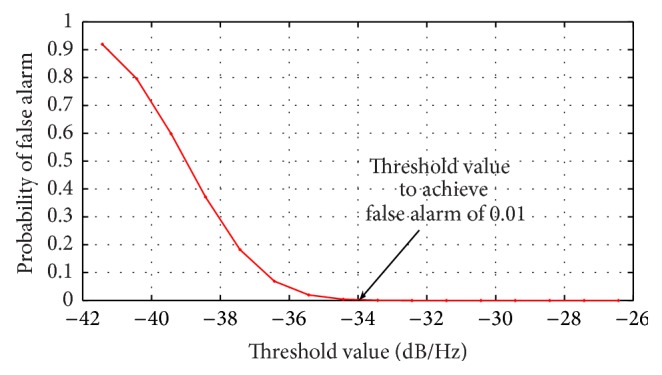
Probability of false alarm for different thresholds using the RVNN based ED in wideband sensing.

An empirical threshold selection method was employed here and *P*_FA_ for each threshold was estimated using (16). This was done by varying the threshold values *V*_*T*_ over the noise PSD and Figure 8 provides the result obtained using the RVNN based ED in wideband sensing. By comparing the threshold line in Figure 6 with Figure 8, it can be easily observed that at a threshold of −35 dB/Hz, less noise samples crossed the threshold (Figure 6) resulting in *P*_FA_ < 0.01 as seen in Figure 8. This provided a suitable threshold for estimating the detector performance.

## 5. Results and Discussion

### 5.1. Varying AR Model Order on RVNN Based ED

**Figure 9:**
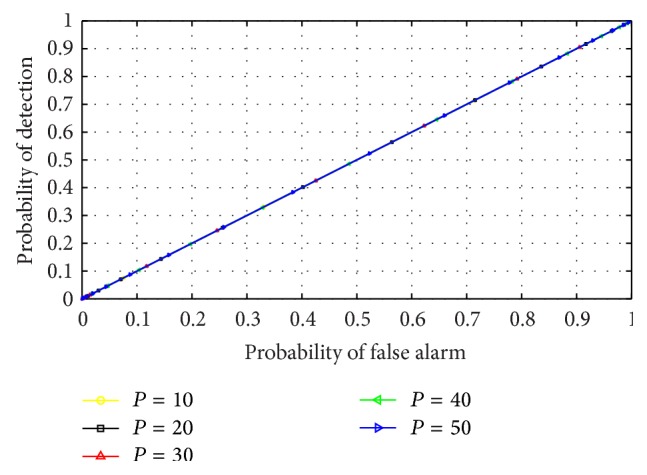
ROC for RVNN based ED in narrowband sensing under low SNR (0 dB) and sample number *N* = 250.

**Figure 10:**
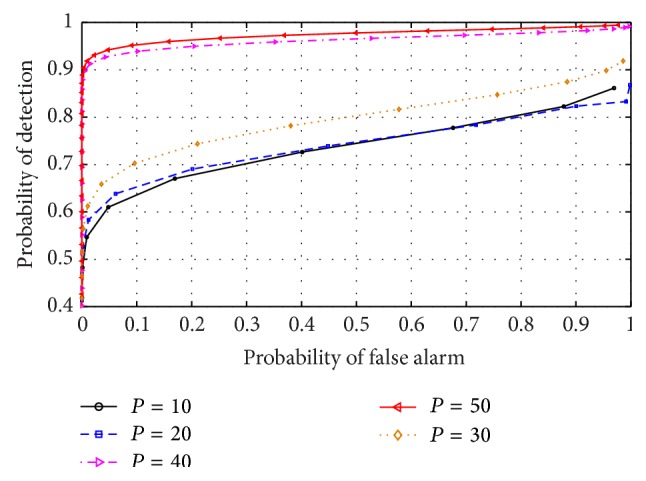
ROC for RVNN based ED in narrowband sensing under high SNR (10 dB) and sample number *N* = 250.

**Figure 11:**
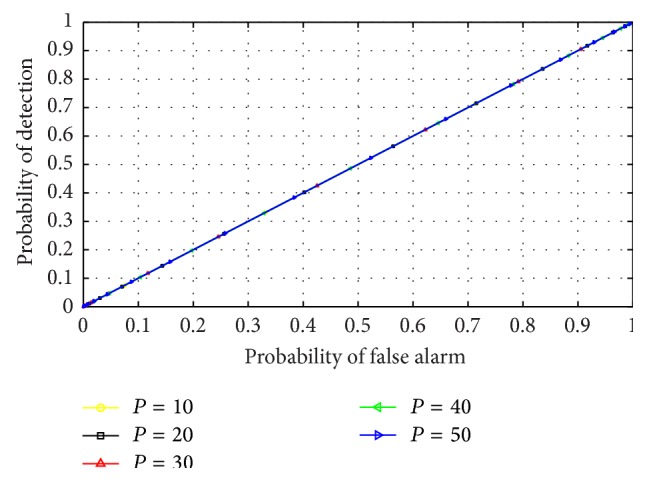
ROC for RVNN based ED in wideband sensing under low SNR (0 dB) and sample number *N* = 250.

**Figure 12:**
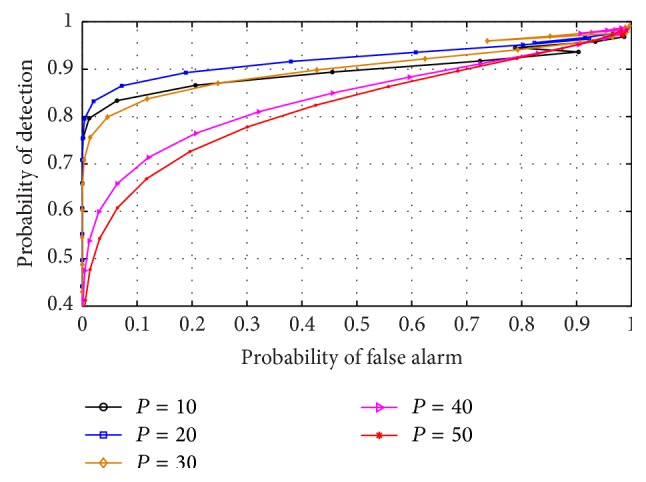
ROC for RVNN based ED in wideband sensing under high SNR (10 dB) and sample number *N* = 250.

The effect of varying AR model order on the performance of the RVNN based ED was analyzed by inspecting the outcome of different ROCs under varying model order values. This examination was essential to aid in the choice of optimum model order for CR operation. Furthermore, this effect was investigated in both narrow and wideband sensing to determine which scenario best supports the detector. The detector was also examined in low (0 dB) and high (10 dB) SNR conditions, respectively. It can be observed from Figures 9 and 11 that the ROCs for different orders remained approximately equal for low SNR in both narrow and wideband sensing. This confirmed that, at low SNR, the PU signal is totally submerged in noise and cannot be differentiated by the use of energy levels alone resulting in *P*_FA_≈*P*_*D*_. This also indicated that irrespective of the approach, the ED typically performs poorly in low SNR conditions. However, in narrowband sensing, high SNR, and varying model order of Figure 10, the RVNN based ED was observed to perform well, particularly for model order *P* = 50. We also observed that the difference in performance becomes practically negligible for *P* ≥ 40. Hence, a minimum order of *P* = 40 was observed to achieve high detection performance. For the case of wideband sensing, high SNR, and varying model order, Figure 12 revealed a reverse in performance for the model order values investigated here. In this case, model order *P* = 50 was observed to perform poorly while *P* = 20 provided better performance. We note that this outcome was based on the increased spectral leakage at lower order which resulted in more samples crossing the threshold than at *P* = 50. This effect is evident owing to the large spectrum considered under wideband sensing as compared to the percentage occupancy of the signal within the band. Hence, it can be inferred that the probability of false alarm would consequently increase for such lower model orders than the higher orders, thus making it uncertain to strictly rely on the results of an ROC curve alone. However, this observation would be further studied and analysed in future works. By comparison, it was observed that narrowband sensing produces better performance than wideband for the RVNN based ED. This can be conclusive if the sensing bandwidth of the narrowband detector corresponds typically to the transmission bandwidth of the PU signal. The implication of these results means that to use the RVNN based ED for CR, narrowband sensing at *P* = 50 for short data length (fast sensing time) will be ideal to produce the best detection performance.

### 5.2. Varying Sample Number on RVNN Based ED

**Figure 13:**
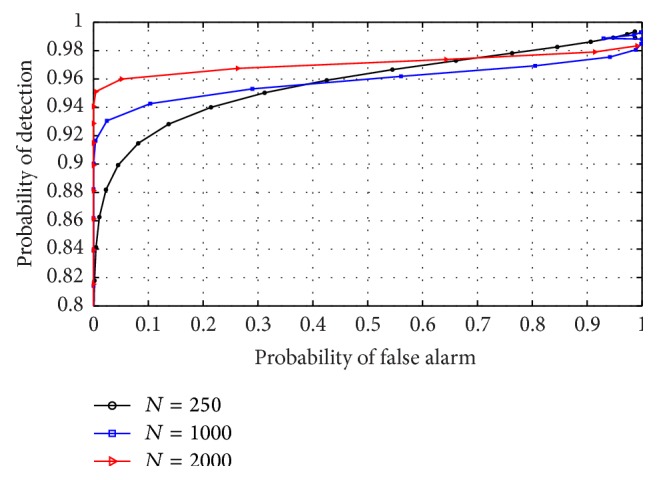
ROC for RVNN based ED in narrowband sensing under high SNR (10 dB) and *P* = 50.

**Figure 14:**
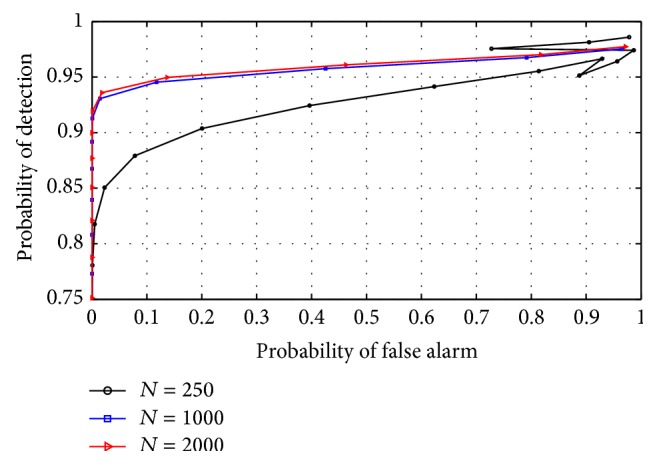
ROC for RVNN based ED in wideband sensing under high SNR (10 dB) and *P* = 20.

To examine this effect, a fixed choice of AR model order *P* = 50 was selected for narrowband sensing while *P* = 20 was chosen for wideband sensing. This ensured that the best model order values were used to examine the effect of sensing time, that is, varying sample number. Here we examined only for the high SNR case (10 dB) knowing that performance remains the same in low SNR conditions (seen in Figures 9 and 11). By observing Figure 13, it was seen that detection performance of RVNN based ED improved with increase in sample number *N*. However, this increase became negligible for *N* ≥ 2000. We note that this performance was achieved for *P* = 50. Furthermore, by using *P* = 20 for wideband sensing, Figure 14 revealed that high performance was sustained even with a slight performance drop in comparison to narrowband sensing. This implied that to use the RVNN based ED for fast sensing in CR, narrowband sensing for *P* = 50 remains ideal.

### 5.3. Comparison with Other Parametric Based ED Techniques

**Figure 15:**
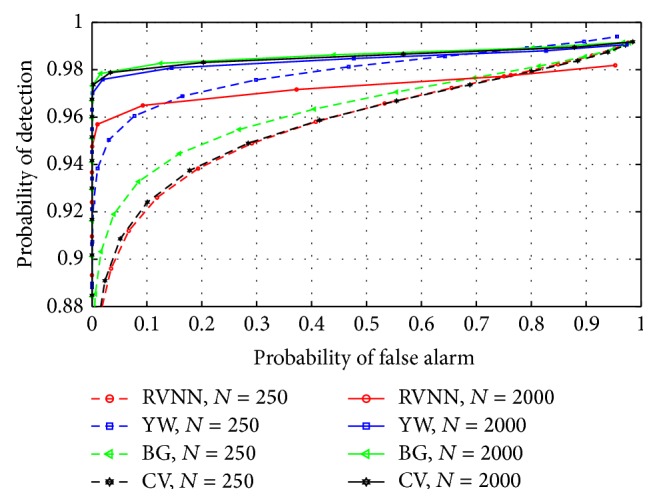
ROCs compared for different parametric based EDs using *P* = 50 in narrowband sensing.

**Figure 16:**
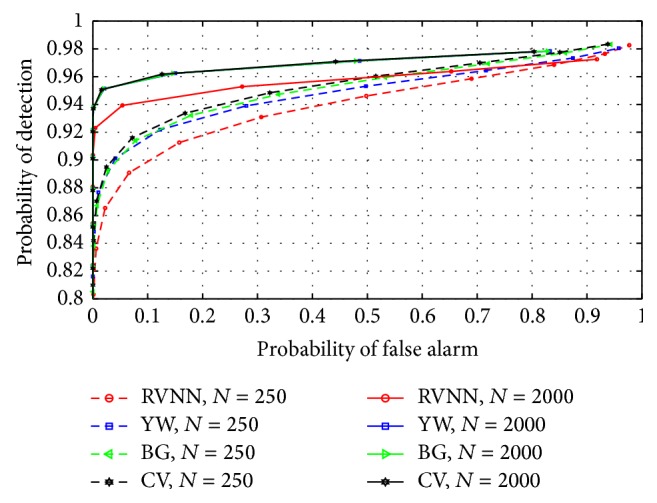
ROCs compared for different parametric based EDs for *P* = 20 in wideband sensing.

**Figure 17:**
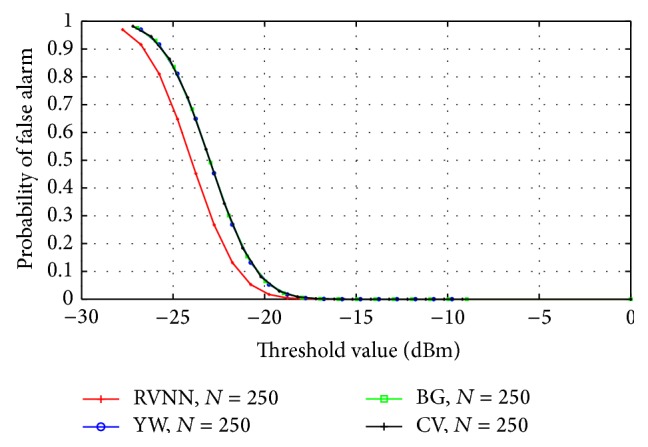
Comparison of probability of false alarm for different parametric based techniques for the case of narrowband sensing at *N* = 250.

The RVNN based ED was compared with the Yule-Walker (YW), Burg (BG), and covariance (CV) based approaches. Narrowband sensing was examined using model order *P* = 50 while *P* = 20 was chosen for wideband sensing.  Figure 15 revealed a close comparative performance between all techniques for fasting sensing time (*N* = 250). Further comparison was conducted for long sensing time (*N* = 2000) and once again, performance level was close. However, it was observed that performance increased for all techniques with increase in sample number. This implied that long sensing time improved the performance of the RVNN based ED and other techniques. For wideband sensing, Figure 16 revealed a close detection performance between RVNN based ED and other parametric techniques; however, a 2% drop in detection performance was observed with respect to BG, YW, and CV. Comparatively, the RVNN based ED produced similar detection performance to acceptable specifications (*P*_*D*_ > 0.9 at *P*_FA_ = 0.1) along with other techniques. In addition, it is shown in Figure 17 that the proposed technique provides a 10% average reduction in *P*_FA_ at thresholds below −20 dBm than YW, BG, and CV. This revealed a possible 1 dB reduction in threshold level to achieve *P*_FA_ = 0.1 as compared to other parametric techniques studied here. This means that the RVNN based ED provides better sensitivity than YW, BG, and CV based approaches. Also, we note that YW, BG, and CV have tendencies to overestimate the magnitude response of a signal which resulted in the observed better ROC performance of YW, BG, and CV over the proposed technique in Figures 15 and 16. Nevertheless, higher SNR estimation and consequently improved ROC can be achieved for the RVNN based ED by including appropriate amplifiers after the square law device in Figure 5. On the other hand, false alarms are more difficult to address and cannot be solved by simple amplification as proposed because both noise and signals will be amplified accordingly, except if noise reduction techniques are used. Consequently, reducing *P*_FA_ could depend more on threshold selection or on the underlying nature of the detector. Therefore, Figure 17 emphasizes a critical advantage of the RVNN based ED over other parametric techniques especially in providing an inherent capacity to reduce the *P*_FA_ level and prevent possible interference to primary user (PU) signals. This is a highly desirable feature of any good detector for CR application.

### 5.4. Comparison with Nonparametric Techniques

**Figure 18:**
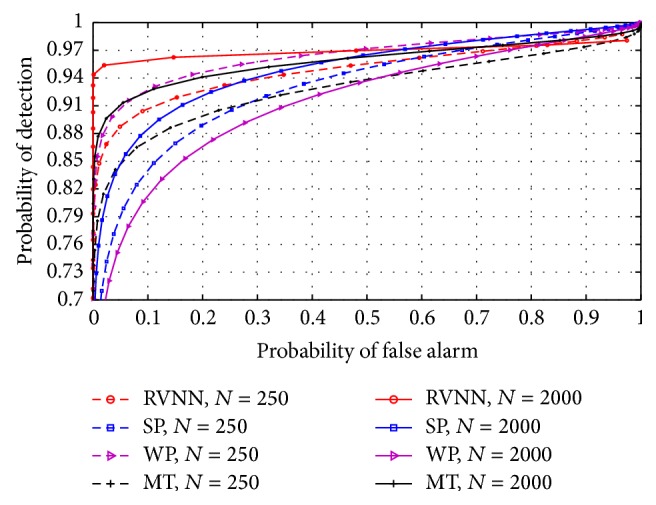
ROCs compared between RVNN based ED and other nonparametric techniques in narrowband for *P* = 50.

**Figure 19:**
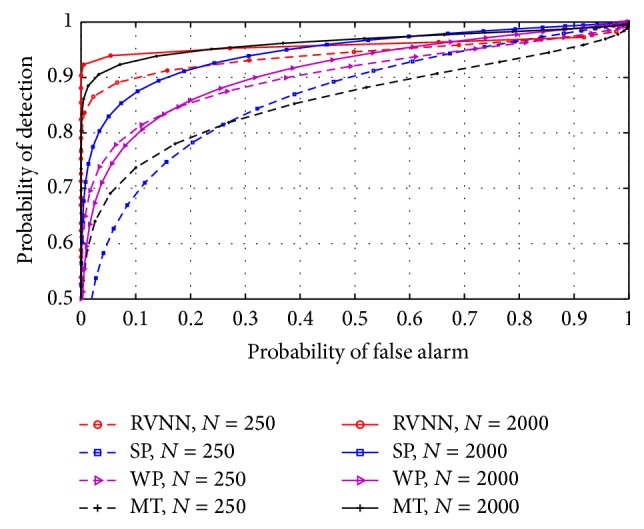
ROCs compared between RVNN based ED and other nonparametric techniques for wideband sensing for *P* = 20.

Finally, the RVNN based ED was compared with nonparametric techniques such as the simple periodogram (SP), Welch periodogram (WP), and multitaper (MT). For narrowband sensing, Figure 18 revealed that the RVNN based ED performed better than the nonparametric techniques compared here except for WP which performed slightly better. This was due to the high variance estimation obtained in the WP which made more noise samples to cross the threshold than in the RVNN based ED. In wideband sensing, Figure 19 revealed that the RVNN based ED performed better than other nonparametric techniques.

## 6. Conclusion

In this paper, a real valued neural network (RVNN) based energy detector (ED) has been proposed, simulated, and analyzed. The choice of the RVNN method for estimating the coefficients of an autoregressive (AR) system was based on its inherent ability to solve the challenge of line splitting and spectral shifting as reported in [23]. Thus, our developed RVNN based ED was examined using different simulation parameters for different sensing conditions. We observed that, for model order *P* = 50, the proposed detector provided a high detection performance in narrowband sensing and similar performance in wideband sensing for *P* = 20. Furthermore, the detector was compared with other parametric based techniques like the Yule-Walker (YW), Burg (BG), and covariance (CV) methods. A close performance was achieved between our proposed ED and others with less than 5% drop in ROC performance observed with respect to YW, BG, and CV. However, a 10% reduction in false alarm was observed for the RVNN based ED over other parametric techniques. This result presents an important advantage in using the proposed technique for CR application because techniques with less false alarms are highly desirable. Finally, it was compared with some nonparametric techniques like the simple periodogram (SP), Welch periodogram (WP), and the multitaper (MT). Our proposed detector provided a 10% detection performance gain over these techniques except in narrowband and fast sensing case where WP provided about 2% detection increase than the RVNN based ED. This was attributed to the high estimated noise variance level which consequently resulted in increased false alarm rates in WP. The study reported here indicates that the RVNN based ED provides a new option for spectrum sensing in CR. However, we note that attention was not given here to the degree of design complexity with respect to choice of higher model order. Though it has been shown in [17] that computational demand in terms of number of multiplications is less than the periodogram, likewise for the storage demand, no closed form model exists to describe complexity for higher model order. This might not necessarily affect detection or false alarm performance but might affect timing performance. However, this remains to be studied and provides an opportunity for future research investigations in the field of AR application in CR.
